# A Flexible Arrayed Eddy Current Sensor for Inspection of Hollow Axle Inner Surfaces

**DOI:** 10.3390/s16070952

**Published:** 2016-06-23

**Authors:** Zhenguo Sun, Dong Cai, Cheng Zou, Wenzeng Zhang, Qiang Chen

**Affiliations:** 1 Department of Mechanical Engineering, Tsinghua University, Beijing 100084, China; caid14@mails.tsinghua.edu.cn (D.C.); zoucme@126.com (C.Z.); wenzeng@tsinghua.edu.cn (W.Z.); chenq@tsinghua.edu.cn (Q.C.); 2 Yangtze Delta Region Institute of Tsinghua University, Jiaxing 314006, China

**Keywords:** eddy current, flexible arrayed sensor, hollow axle, frequency optimization

## Abstract

A reliable and accurate inspection of the hollow axle inner surface is important for the safe operation of high-speed trains. In order to improve the reliability of the inspection, a flexible arrayed eddy current sensor for non-destructive testing of the hollow axle inner surface was designed, fabricated and characterized. The sensor, consisting of two excitation traces and 28 sensing traces, was developed by using the flexible printed circuit board (FPCB) technique to conform the geometric features of the inner surfaces of the hollow axles. The main innovative aspect of the sensor was the new arrangement of excitation/sensing traces to achieve a differential configuration. Finite element model was established to analyze sensor responses and to determine the optimal excitation frequency. Experimental validations were conducted on a specimen with several artificial defects. Results from experiments and simulations were consistent with each other, with the maximum relative error less than 4%. Both results proved that the sensor was capable of detecting longitudinal and transverse defects with the depth of 0.5 mm under the optimal excitation frequency of 0.9 MHz.

## 1. Introduction

High-speed railways integrating a number of advanced rail technologies have been developed in many countries for passenger transportation and freight services, and they have become increasingly popular around the world due to their convenient, efficient, reliable and comfortable features. Axles are key components which are subject to dynamic loads in bogie systems, and may directly affect the safety of train operations. The traditional solid axles have been replaced by hollow axles to decrease the unsprung mass and consequently reduce the forces between rails and wheels. Failures and defects caused by material, processing, assembling, fatigue loading and complicated operating environments, can not only be observed on the outer surfaces of the hollow axles, but also on the inner surfaces. Thus, inspection of the inner surfaces of hollow axles plays a key role in the quality inspection process of hollow axles.

So far, several non-destructive testing (NDT) techniques have been designed and developed for the inspection of hollow axles in practice. The ultrasonic testing (UT) technology has been widely used in the in-service NDT equipment to monitor hollow axles. Several ultrasonic transducers with different incident angles were arranged in a probe holder, which could rotate and move within a hollow axle, enabling the detection of transverse (circumferential) and longitudinal (axial) defects at different locations of the hollow axle covering both inner surfaces and outer surfaces [1,2]. The ultrasonic phased array technique has also been used in inspection of hollow axles along with the synthetic aperture focusing technique to improve the lateral resolution and testing efficiency [3,4]. In spite of the high thickness-to-wavelength ratio, Li et al. [5] designed a guided wave-based structural health monitoring method for damage detection of hollow axles. Another inspection method using guided wave phenomenon in combination with a modified pulse-echo approach was presented by Ziaja et al. [6] to detect the cracks within the specific sections of a hollow axle. Ultrasonic inspection techniques play a dominant role in flaw detection of the hollow axle outer surface. However, UT is not sufficiently sensitive to identify shallow defects on the inner surfaces of axels due to the existence of natural waves, caused by the interface between the coupling liquid and the hollow axle, leading to the issue of false-calls [7]. Cavuto et al. [8] proposed a laser-ultrasonic technique for the inspection of the hollow axle outer surface. An air-coupled ultrasonic probe was utilized to detect the ultrasonic waves generated by a high-power pulsed laser. However, no inspection application on the inner surface was demonstrated. Induction thermography is an alternative method with high detection sensitivity and high testing speed for the outer surface inspection of a hollow axle [4], yet no inspection application on inner surfaces of hollow axles was mentioned. The electromagnetic method, such as eddy current testing, can work as an alternative to address the limitations of UT. Chady et al. [9] designed two kinds of probes based on flux leakage and eddy current, respectively, for the inspection of the hollow axle inner surface. One of the configuration consisted of the permanent magnet for magnetizing the ferromagnetic axle and a matrix of anisotropic magneto resistive three axis sensors. The other one used excitation coil to generate electromagnetic field which was picked up by Hall effect sensors. An integrated electromagnetic testing system combining eddy current testing with magnetic memory testing was developed to detect cracks and stress concentrations at the inner surfaces of hollow axles [7].

Flexible arrayed eddy current sensors, which are suitable for the inspection of components with complex geometries, are becoming a research hotspot [10,11,12]. Crouch et al. [13] used a flexible printed board with multiple eddy-current coil pairs to produce a rapid mapping of the external pipeline corrosion. Flexible arrayed eddy current sensors have also been applied in measurements of bond coat and top coat material properties and thicknesses, such as the flexible Meandering Winding Magnetometer array presented in [14]. Endo et al. [15] proposed a flexible arrayed eddy current probe with several coil pairs and a 12-decibel drop method for crack length evaluation. Another flexible arrayed eddy current sensor for condition-based maintenance of key components of aircraft was presented in [16] where the sensor was comprised of 64 elements with resolution of 0.8 mm and an algorithm used for sizing the crack length based on the sensor was also presented.

A new flexible arrayed eddy current sensor for the inspection of the hollow axle inner surface is proposed in this paper. The design and the principle of the sensor are presented in Section 2. The validation of the sensor behaviors and the optimization of the excitation frequency by simulation and experiments are introduced subsequently.

## 2. Sensor Design

Differential-mode eddy current probes are more sensitive to small discontinuities and have the advantage of suppressing lift-off effect, and thus have been widely used in eddy current testing [17]. Therefore, a flexible arrayed eddy current sensor in a differential mode, characterized by high detection efficiency due to multiple sensing elements, was designed to inspect the hollow axle inner surface. In order to obtain higher signal-to-noise ratio and better detection sensitivity, the sensor was configured as transmit/receive type. Copper traces in FPCB were used to form the excitation elements and the sensing elements, to guarantee the flexibility and consistency of the sensors.

**Figure 1:**
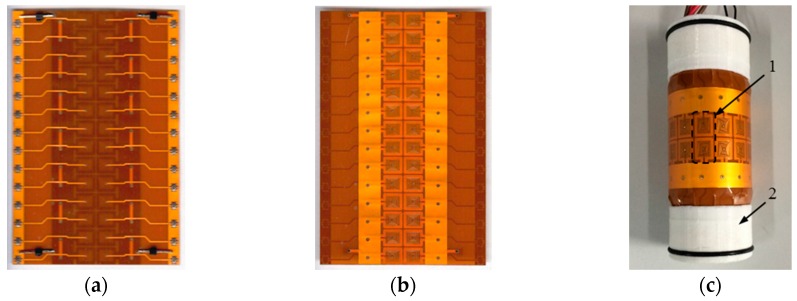
Novel flexible arrayed sensor (**a**) Bottom view of the unfolded sensor; (**b**) Top view of the unfolded sensor; (**c**) Actual sensor; 1—Differential sensing pair, 2—Sensor holder.

**Figure 2:**
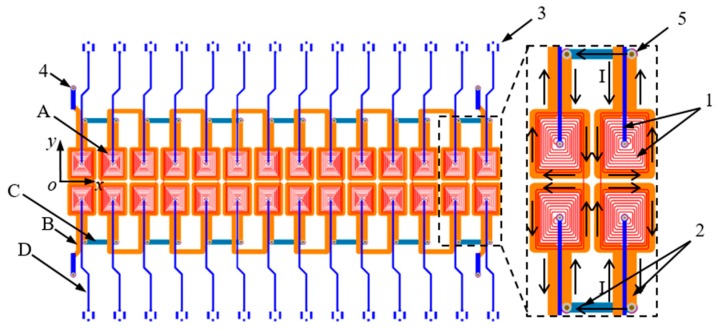
Schematic diagram of the sensor. A—Top layer; B—Mid-layer 1; C—Mid-layer 2; D—Bottom layer; 1—Sensing traces; 2—Excitation traces; 3—Terminals of sensing traces; 4—Terminals of excitation traces; 5—via hole.

The four-layered FPCB with excitation traces and sensing traces was rolled and mounted on the sensor holder with 28.6 mm in diameter as shown in Figure 1 and Figure 2. The sensor is symmetric about the x axis to work in the differential configuration. There are two independent excitation traces with the same alternating current flowing in the direction shown in Figure 2. The sensor consists of 28 sensing traces, among which every two adjacent traces along y axis are connected to function as a differential sensing pair. Each of the sensing elements has nine windings which are surrounded by the excitation trace. Adjacent sensing traces along x axis and y axis are winded in the opposite direction. Excitation traces are 1 mm in width while the width of sensing traces is 0.1 mm with gaps of 0.2 mm between windings, and the element spacing is 6.5 mm.

When an alternate current I flows in excitation traces, an alternating magnetic field B is generated, which induces a voltage on each of the sensing traces given by:

(1) $$V_{t}=-\sum_{i\;=\;1}^{N}\frac{d\phi_{t,W_{i}}}{dt}$$

where subscript *t* denotes the sensing trace number, *N* is the number of windings, and $\phi_{t,W_{i}}$ is the magnetic field flux depending on the area defined by each winding $W_{i}$. For each of the differential sensing pairs, the output voltage $V_{out}$ can be expressed as:

(2) $$V_{out}=\left(-\sum_{i\;=\;1}^{N}\frac{d\phi_{1,W_{i}}}{dt}\right)+\left(-\sum_{i\;=\;1}^{N}\frac{d\phi_{2,W_{i}}}{dt}\right)=-\sum_{i\;=\;1}^{N}\frac{d\left(\phi_{1,W_{i}}+\;\phi_{2,W_{i}}\right)}{dt}$$

where subscripts 1 and 2 denote two sensing traces of a differential sensing pair, respectively. If sensing traces of a differential sensing pair are under the same circumstance, the induced current on sensing traces will be of the same magnitude, but the output voltage will remain close to zero due to the winding direction of the sensing traces and the flow direction of the excitation current. Otherwise, the voltage contribution of a sensing trace will not be canceled by the contribution of the other sensing trace leading to nonzero output voltages.

## 3. Finite Element Modeling

**Figure 3:**
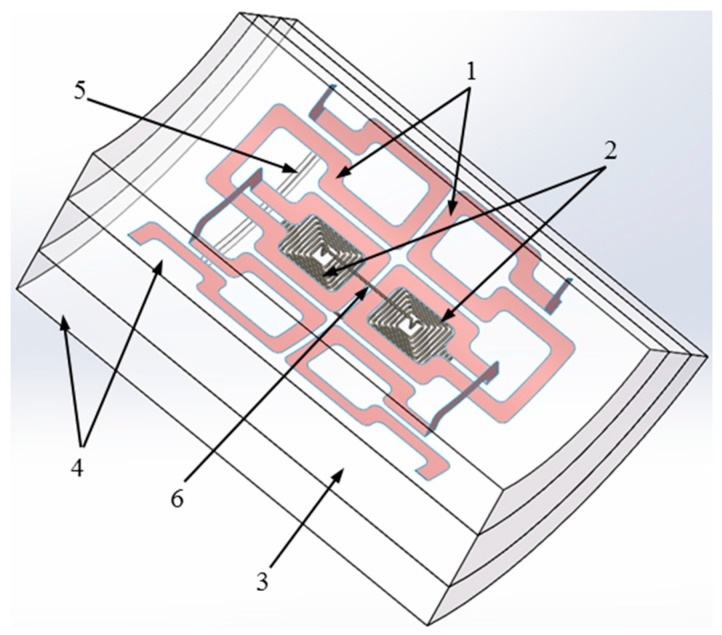
Simulation model of the sensor. 1—Excitation traces; 2—Sensing traces; 3—Segment of hollow axle; 4—Air; 5—Artificial Defect; 6—Lumped element.

The proposed sensor was simulated using a finite element modeling (FEM) software, COMSOL Multiphysics, to validate the sensor behaviors and optimize the excitation frequencies. To simplify the simulation model, only excitation traces and a differential sensing pair of the sensor were included in the model with the hollow axle, the artificial defect and the air as shown in Figure 3. The air gap between the sensing traces and the hollow axle inner surface was 0.5 mm. A Lumped Element node was used to mimic the insertion of a resistor between output boundaries of a differential sensing pair to measure the output voltage generated by eddy current. Excitation traces were modeled as Single-Turn Coil nodes subjected to a current excitation. Only a small segment of the hollow axle was simulated at the frequency domain with the consideration of time consumption and hardware requirement. The Magnetic Insulation interface was applied at all of the outer boundaries of the model, which set the tangential components of the magnetic potential to zero at these boundaries. The Free Tetrahedral node was added to create an unstructured tetrahedral mesh with a minimum element size of 0.048 mm and a maximum element growth rate of 1.35. The hollow axle steel and the air were applied in the domain 3 and 4, respectively. The electrical conductivity of the hollow axle steel (ASTM 1050) is 5.655 × 10^6^ S/m. Both excitation traces and sensing traces were defined as copper. The domain of the artificial defect was considered as the air.

**Figure 4:**
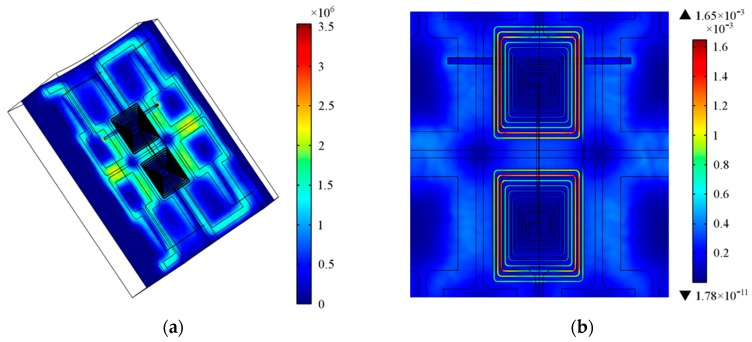
(**a**) Distribution of the eddy current density (A/m^2^), f = 1MHz; (**b**) Distribution of the magnetic flux density (T), f = 1 MHz.

At first, the model was simulated at 1 MHz with a defect located under one of the sensing traces. The distribution of the eddy current density in the hollow axle steel is shown in Figure 4a. As can be seen, eddy current loops, generated at the inner surface of the hollow axle, are similar to the shape of excitation traces. The current density of regions below two adjacent segments of excitation traces is much higher since the excitation current of these adjacent segments is of the same direction. The existence of defect changes the distribution of the eddy current, making the eddy current concentrate on two ends of the defect. These features can also be observed by the distribution of the magnetic flux density as shown in Figure 4b.

**Figure 5:**
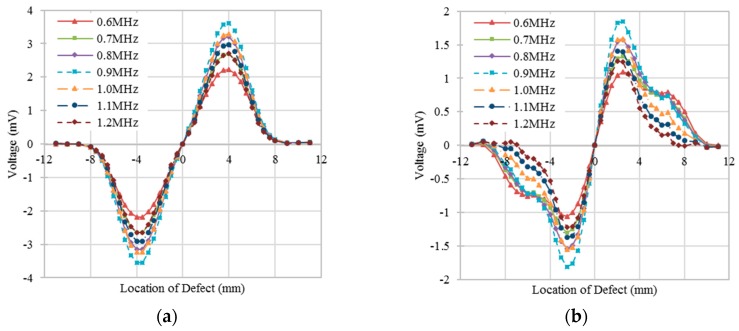
Output voltage of a differential sensing pair with the position sweep of defects at different excitation frequencies. (**a**) Transverse defect with 0.5 mm in depth; (**b**) Longitudinal defect with 0.5 mm in depth.

**Figure 6:**
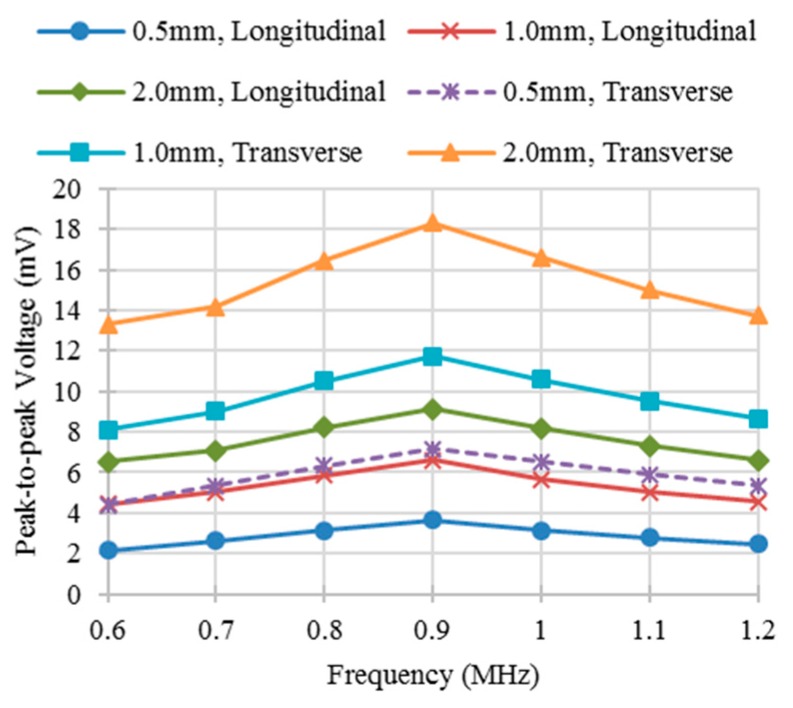
Peak-to-peak voltage for different kinds of defects at different frequencies from simulation. (“0.5 mm, Longitudinal” denotes longitudinal defect with 0.5 mm in depth).

To obtain the sensor response at different excitation frequencies, the defect was positioned at different locations relative to the sensor. Figure 5a,b shows the change of the output voltage of a differential sensing pair for different excitation frequencies and positions of a transverse defect and a longitudinal defect, respectively. Limited difference of the shape of curves for these two kinds of defects with the same defect depth was observed because of the different defect orientations. The peak-to-peak values of the output voltages were measured to determine the optimal excitation frequency as shown in Figure 6. Figure 6 shows when the excitation frequency is 0.9 MHz, the best response can be obtained for all of the simulated defects.

## 4. Experimental Validation

### 4.1. Experimental Set-up

**Figure 7:**
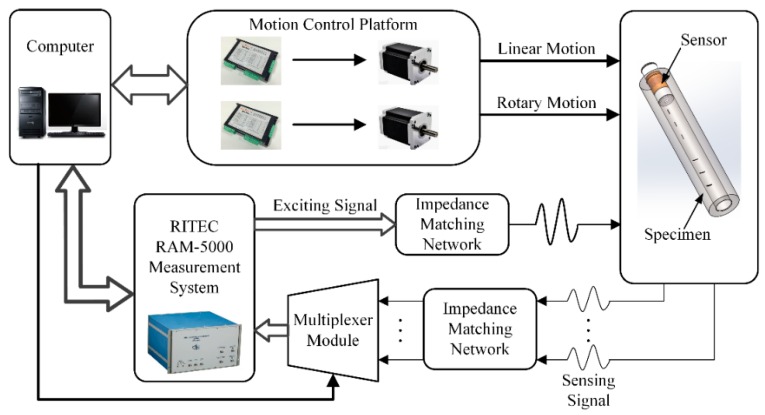
Diagram block of the experimental system.

An experimental system was set up to validate the simulation results and the feasibility of the sensor as shown in Figure 7. The motion control platform consisted of stepper motors, stepper motor drivers and a corresponding transmitting module to implement the linear and rotary motions of the sensor. The generation of the excitation signal and detection of the differential output voltage were realized by a RITEC RAM-5000 measurement system, which featured a broadband gated RF amplifier, a unique tracking receiver, quadrature phase sensitive detectors, gated integrators and multiple frequency synthesizers. Since the measurement system had only two reception channels, a multiplexer module was designed for the reception of output voltages of 14 differential sensing pairs. Both the excitation and the reception channels were configured with the impedance matching network to make the status of excitation and reception at their best. Processed data was transmitted to a PC with a LabVIEW interface for inspection configuration, motion control and results visualization.

Experiments were conducted by using a hollow axle specimen made of ASTM 1050 steel. Both longitudinal and transverse artificial defects with depths of 0.5 mm, 1.0 mm and 2.0 mm studied in the FEM simulation were reproduced on the specimen by electro-erosion. All defects are 0.35 mm in width and 10 mm in length. A differential sensing pair was activated to measure the sensor responses at different excitation frequencies for verification of simulation results. The inspection of the specimen was carried out by using all the differential sensing pairs with the axial and circumferential sampling intervals of 1 mm and 1.29°, respectively. In order to achieve the resolution, the sensor needed to rotate 20 times with a rotation angle of 1.29° after each axial step with a step distance of 1 mm and then rotated reversely after next axial step with the same motion parameters. The excitation and reception of the sensor were implemented after each rotation. The gain of the RITEC RAM-5000 measurement system was set as 13 dB.

### 4.2. Experimental Results

**Figure 8:**
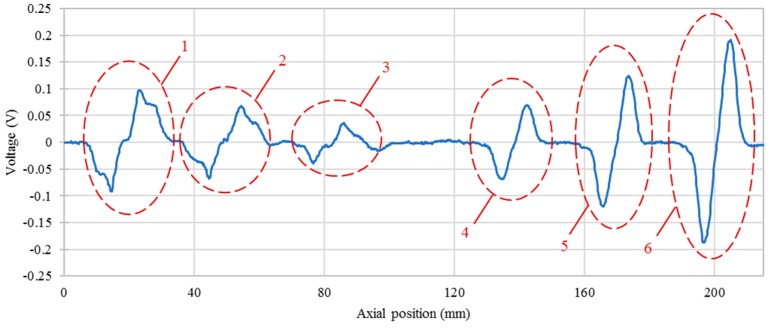
Output voltage of a differential sensing pair along a linear sweep of the sensor position at 0.9 MHz. 1—2.0 mm, longitudinal defect; 2—1.0 mm, longitudinal defect; 3—0.5 mm, longitudinal defect; 4—0.5 mm, transverse defect; 5—1.0 mm, transverse defect; 6—2.0 mm, transverse defect.

**Figure 9:**
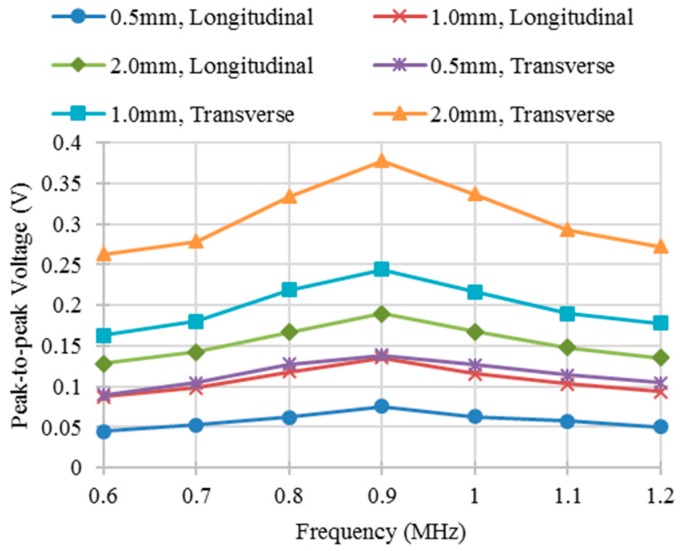
Peak-to-peak voltage for different kinds of defects at different frequencies from experiment.

Figure 8 shows the change of the output voltage for a linear sweep of the sensor position through the whole specimen. When the first sensing trace of the differential sensing pair approaches a defect, the output voltage decreases from zero to its minimum. Then it starts to increase and approximates to zero when the center of the pair is aligned with the center of the defect. Subsequently, the voltage increases and then decreases as the sensor leaves the defect. The shapes of the curves for longitudinal and transverse defects are different, which is consistent with the simulation results. The peak-to-peak values of the output voltages for different kinds of defects and different excitation frequencies were obtained experimentally as shown in Figure 9. The peak-to-peak voltage achieves the maximum value when the excitation frequency is 0.9 MHz which is the same as the one mentioned in simulation section above. The corresponding values from experiments and simulations are different because the voltage values from experiments were pre-amplified by the RITEC RAM-5000 measurement system while the voltage values from simulations were measured directly from two ends of the sensing trace. In order to quantitatively compare results from experiments and simulations, the results from simulations were multiplied by the gain used in the experiments and then the relative errors were calculated as shown in the Table 1. The maximum relative error is less than 4%.

**Figure 10:**
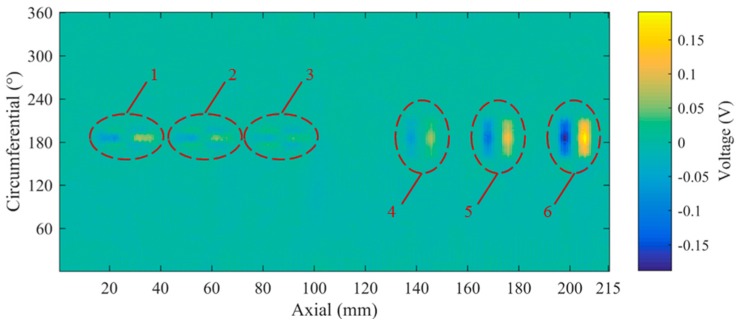
Inspection result of the specimen. 1—2.0 mm, longitudinal defect; 2—1.0 mm, longitudinal defect; 3—0.5 mm, longitudinal defect; 4—0.5 mm, transverse defect; 5—1.0 mm, transverse defect; 6—2.0 mm, transverse defect.

The inspection of the specimen was conducted at 0.9 MHz. All of the 6 artificial defects were detected as shown in Figure 10, where the maximum noise amplitude is 0.012 V and the response amplitude of longitudinal defect with 0.5 mm depth is 0.038 V. The defect signal amplitude is three times higher than the noise amplitude, which proves the feasibility of the proposed sensor. The noise mainly comes from electronic noises of the RITEC RAM-5000 measurement system and lift-off effect. The latter one is the dominant factor resulting from the processing error of the hollow axle inner surface and the sensor holder. As can be noticed, the sensor is more sensitive to the transverse defects.

## 5. Conclusions

A flexible arrayed eddy current sensor has been presented. This new design is suitable for the inspection of the hollow axle inner surfaces. The optimal excitation frequency, which is 0.9 MHz, is determined by FEM simulation, whose results are in good agreement with the experimental results. The maximum relative error between simulations and experiments is less than 4%. Results from simulations and experiments show the sensor is capable of detecting both longitudinal and transverse defects with depths as small as 0.5 mm. The sensor is more sensitive to the transverse defects, therefore future work is required to increase the sensibility of the sensor to the longitudinal defects.

## Figures and Tables

**Table 1:** The relative errors of results from experiment and simulation (%).

	Frequency (MHz)	0.6	0.7	0.8	0.9	1.0	1.1	1.2
Defect Type								
2.0 mm, longitudinal		1.79 ^1^	0.95	1.58	3.79	2.06	1.35	2.64
1.0 mm, longitudinal		2.14	2.23	0.75	2.20	2.17	2.23	2.71
0.5 mm, longitudinal		2.72	0.76	0.53	2.59	0.07	2.84	1.05
2.0 mm, transverse		1.28	1.54	1.43	3.14	1.35	2.38	0.76
1.0 mm, transverse		0.23	0.11	3.84	3.95	2.15	0.23	2.56
0.5 mm, transverse		1.59	2.29	0.31	3.44	2.83	2.84	2.06

^1^ The relative errors are calculated using $\delta \text{ = }\left|V_{pp,Exp}-V_{pp,Sim}\right|/V_{pp,Exp}\times 100\text{\%}$, where $V_{pp,Exp}$ denotes the peak-to-peak voltage for different kinds of defects at different frequencies from experiments, $V_{pp,Sim}$ the peak-to-peak voltage for different kinds of defects at different frequencies from simulations.
